# Prevalence and diversity of gastrointestinal protozoa in Madura cattle at Bangkalan Regency, East Java, Indonesia

**DOI:** 10.14202/vetworld.2019.198-204

**Published:** 2019-02-08

**Authors:** Poedji Hastutiek, Wiwik Misaco Yuniarti, Mufasirin Djaeri, Nunuk Dyah Retno Lastuti, Endang Suprihati, Lucia Tri Suwanti

**Affiliations:** 1 Department of Parasitology, Faculty of Veterinary Medicine, Universitas Airlangga, Jl. Mulyorejo, Kampus C Unair, Surabaya, Indonesia; 2 Department of Clinical Science, Faculty of Veterinary Medicine, Universitas Airlangga, Jl. Mulyorejo, Kampus C Unair, Surabaya, Indonesia; 3 Department of Parasitology, Faculty of Veterinary Medicine, Universitas Airlangga, Institute of Tropical Diseases, Universitas Airlangga, Jl. Mulyorejo, Kampus C Unair, Surabaya, Indonesia

**Keywords:** Bangkalan, Madura cattle, mapping, protozoa

## Abstract

**Aim:**

This study aimed to describe the gastrointestinal protozoa in Madura cattle at Bangkalan Regency, East Java, Indonesia.

**Materials and Methods:**

A total of 500 samples of Madura cattle feces were collected from 10 districts at Bangkalan Regency. Those ten districts represent the lowland and upland areas, and each district was represented by one village. The collected feces were examined using native, sedimentation, and floating methods. The species identification was determined by their morphology.

**Results:**

There were 357 (71.4%) samples positively infected with protozoan. The highest rate of sample with protozoan infection was at Kamal District (88.23%), and Bangkalan District (52.83%) was the lowest one. There were six species of protozoa that infected gastrointestinal tract; those are *Eimeria* spp., *Balantidium* spp., *Isospora* spp., *Blastocystis* spp., *Entamoeba* spp., and *Cryptosporidium* spp. The highest number of protozoa found in this research was *Eimeria* (53.42%) followed by *Blastocystis* (14.43%). In this study, we found that 295 samples (58.76%) infected by one kind of protozoa, 53 samples (10.56%) infected by two kinds of protozoa, and 11 samples (2.19%) infected by three kinds of protozoa. In addition, there were 65.54% of bulls infected with protozoa, considerably lower than cows (72.97%). Cattle aged 6 months-2 years old (73.39%) and >2 years old (71.25%) are known more prone to protozoan infections than cattle aged <6 months (66.15%).

**Conclusion:**

The present study revealed that protozoan infection of cattle is common in Bangkalan Regency. Studies focused on determining that the prevalence of protozoan, risk factors for the parasitism, and the geographic distribution are needed and will be effective guide for prevention and control measures.

## Introduction

Madura cattle is one of the Indonesian local cattle that are widely developed in East Java, especially in Madura Island. Madura cattle have great potentials to be developed because they are genetically tolerant to hot climates and marginal environments, resistant to tick infestations, and highly adaptable to low feed quality, as well as require less food compared to imported cattle. Besides, Madura cattle are easy to maintain, easy to breed, and resistant to various diseases [1]. The population of Madura cattle in Bangkalan Regency in 2016 was 200,279 and predicted to increase every year parallel to the promotion of crossbreed artificial insemination program between Madura cattle and Limousine cattle (Madrasin) [2].

Beef cattle as a potential commodity in the development of rural farming must be supported by an adequate maintenance system. The business of animal husbandry is faced with problems of reproductive disorders and chronic parasitic diseases, especially protozoan infections. Gastrointestinal disease needs special attention because it can be an obstacle that affects the acceleration of livestock development in the countryside so that it can cause economic losses due to decreased livestock productivity, decreased weight, quality of meat, skin, and internal organs, growth retardation in young animals, and danger of zoonoses. Delay weight gain in cattle with protozoan infection can reach >40% compared with healthy cows [3,4].

Based on the examination results of fecal samples gathered from Madura cattle butchered at slaughterhouse at Surabaya, it was found that those cattle were infected by several kinds of protozoa species, such as *Eimeria* spp., *Balantidium* spp., and *Entamoeba* spp. (unpublished data). However, there has been no research published about protozoan infections in the gastrointestinal tract of Madura cattle in Bangkalan Regency, Madura, Indonesia.

## Materials and Methods

### Ethical approval

The present study was based on the laboratory examination of cattle feces without treatment. The samples were collected as per standard sample collection procedure, directly from the rectum without disturbing the animals, and were accompanied by a responsible veterinarian.

### Study area

This research was conducted at 10 districts located in coastal areas (each district was represented by one village) in Bangkalan Regency with high livestock population. Fecal sampling was conducted at sites with different altitudes. Bangkalan, Socah, and Kamal districts are located on lowland with altitude <25 m above sea level, while Tanjung Bumi, Sepuluh, Klampis, Arosbaya, Burneh, Tragah, and Labang districts are located on a plateau with an altitude of 25-200 m above sea level.

### Fecal sample collection and analysis

A total of 500 fecal samples were collected for this research. Fecal samples were taken randomly from bulls and cows and then divided based on age such as <6 months, 6 months-2 years, and >2 years. Fecal sampling was conducted from April to May 2017. During fecal sampling process, questionnaire and interview with the farmer were also conducted to obtain certain information about farmer (name, age, gender, education, and breeding experience) and cattle (breed and quantity, gender, age, type of maintenance, treatment that has ever given, the other reared cattle, type or material of the enclosure, environmental condition, or livestock care).

The samples were collected directly from the rectum and brought to the laboratory in mini zip locked polythene bags and added with 2.5% potassium bichromate for examination. Each plastic bag was labeled with registered sample number and then stored in a container with ice. Afterward, the fecal samples were examined using native methods, as well as sedimentation and modified Fulleborn’s floating methods [5]. To determine the existence of protozoa, identification key methods were carried out [6]. The positive result of protozoan-infected cattle could be determined when protozoa were found during examination using one of those methods. The prevalence of the protozoan infections was expressed in percentage value using the following formula:

P = (Positive results: Number of samples) × 100%.

### Statistical analysis

The data obtained will be analyzed descriptively and presented in the form of the prevalence of protozoan infections based on district, kind of protozoa, sex, and age.

## Results and Discussion

Based on this research, it was found that there were 357 from 500 samples (71.4%) positively infected by protozoa. The highest prevalence of protozoan infections was found in Kamal district, in which 88.23% (45/51) samples were positive, followed by Sepuluh district 86.8% (46/53), Klampis district 80% (32/40), Tanjung Bumi district 75% (36/48), Arosbaya district 74.51% (38/51), Burneh district 68% (34/50), Labang district 67.31% (35/52), Socah district 67.28% (37/55), Tragah district 57.14% (28/49), and Bangkalan district 52.83% (28/53) (Table-1).

**Table-1:** The prevalence of protozoan infection in the gastrointestinal of Madura cattle in each district in Bangkalan Regency.

District	Number of samples	Number of positive samples (%)
Mangkon Arosbaya	51	38 (74.51)
Bator Ma’adan Klampis	40	32 (80)
Keleyan Socah	53	35 (66.04)
Gili Anyar Kamal	51	45 (88.24)
Maneron Sepuluh	53	46 (86.79)
Bumi Anyar Tanjung Bumi	48	36 (75)
Bringen Labang	52	35 (67.31)
Kemoneng Tragah	49	28 (57.14)
Ketengan Burneh	50	34 (68)
Kramin Bangkalan	53	28 (52.83)
Total	500	357 (71.4)

The high prevalence in this study was in accordance with the study of Volpato *et al*. [7], which reported the prevalence of intestinal protozoan infection in dairy calf in Brazil. Those ten districts are located in coastal areas and are well known as livestock meeting points from several locations as well as temporary shelters for cattle that will be traded out of the island or for Idul Adha. On the other hand, the results of this research indicate that the prevalence of protozoan infections in Madura cattle from those 10 districts was high. Therefore, routine monitoring for protozoan infections should be preceded by performing fecal examination so that the infections can be completely controlled to improve the health and productivity of the cattle.

Research result showed that, from 359 positive samples, there were 295 (58.76%) samples infected by one kind of protozoan, 53 (10.56%) samples by two kinds of protozoan, and 11 (2.19%) samples infected with three kinds of protozoan.

The highest of single species protozoan infection was at Klampis district 80% (32/40), while the lowest was at Bangkalan district 49.06% (26/53). District with the highest infection of two species protozoan was Sepuluh district with 26.42% (14/53), whereas the lowest one at Bangkalan district with only 3.77% (2/53). At Kamal district, there were 9.8% (5/51) cattle infected with three kinds of protozoan species. At Labang district, Arosbaya district and Socah district were 7.69% (4/52), 1.96% (1/51), and 1.82% (1/55) respectively (Table-2).

**Table-2:** The prevalence of protozoan species in the gastrointestinal tract of Madura cattle in Bangkalan Regency.

Districts	Samples positively infected with protozoa						Number of positive samples (%)

	One type of protozoan species	Total	Two types of protozoan species	Total	Three types of protozoan species	Total	
Mangkon Arosbaya	*Eimeria* *Balantidium*	31 1	*Eimeria* *Balantidium*	5	*Eimeria* *Balantidium Isospora*	1	38 (74.51)
Bator Ma’adan Klampis	*Eimeria*	32	-		-		32 (80)
Keleyan Socah	*Eimeria*	33	*Eimeria* *Blastocystis*	1	*Eimeria* *Blastocystis* *Balantidium*	1	35 (66.04)
Gili Anyar Kamal	*Eimeria*	12	*Eimeria* *Balantidium*	2	*Eimeria* *Blastocystis* *Balantidium*	3	45 (88.23)
	*Blastocystis*	14	*Eimeria* *Blastocystis*	9	*Eimeria* *Blastocystis* *Entamoeba*	2
	*Balantidium*	2	*Blastocystis Balantidium*	1		
Maneron Sepuluh	*Eimeria*	22	*Eimeria* *Balantidium*	2	-	-	46 (86.8)
	*Blastocystis*	10	*Eimeria* *Blastocystis*	11			
			*Blastocystis* *Balantidium*	1			
Bumi Anyar Tanjung Bumi	*Eimeria*	23	*Eimeria* *Blastocystis*	5	-	-	36 (75)
	*Blastocystis*	7					
	*Balantidium*	1					
Bringen Labang	*Eimeria*	10	*Eimeria* *Blastocystis*	3	*Eimeria* *Blastocystis* *Entamoeba*	1	35 (67.31)
	*Blastocystis*	14	*Blastocystis* *Balantidium*	1	*Eimeria* *Blastocystis* *Balantidium*	1	
	*Entamoeba*	1	*Blastocystis* *Entamoeba*	1	*Eimeria* *Blastocystis* *Cryptosporidium*	1	
			*Blastocystis Cryptosporidium*	1	*Eimeria* *Blastocystis* *Isospora*	1	
Kemoneng Tragah	*Eimeria*	12	*Eimeria* *Blastocystis*	5	-	-	28 (57.14)
	*Blastocystis*	11					
Ketengan Burneh	*Eimeria*	13	*Eimeria* *Balantidium*	2	-	-	34 (68)
	*Balantidium*	18	*Eimeria* *Entamoeba*	1			
Kramat Bangkalan	*Eimeria*	21	*Eimeria* *Balantidium*	2	-	-	28 (52.83)
	*Balantidium*	4					
	*Blastocystis*	1					

**Figure-1:**
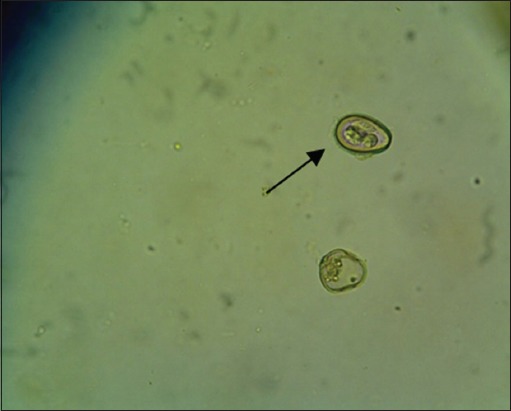
*Blastocystis* spp. (400×).

**Figure-2:**
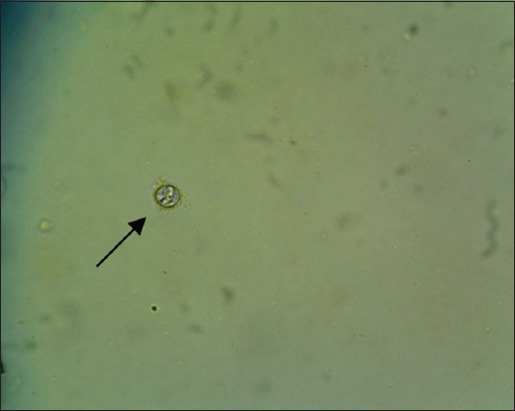
*Cryptosporidium* spp. (400×).

**Figure-3:**
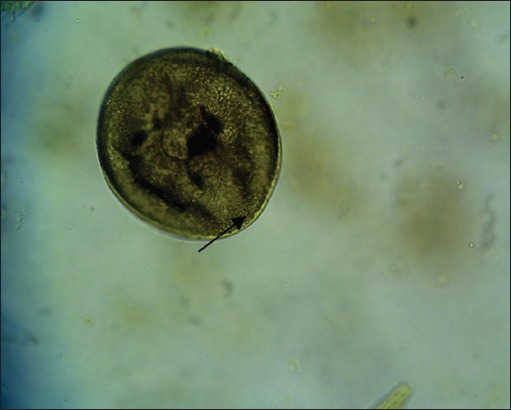
*Balantidium* (400×).

**Figure-4:**
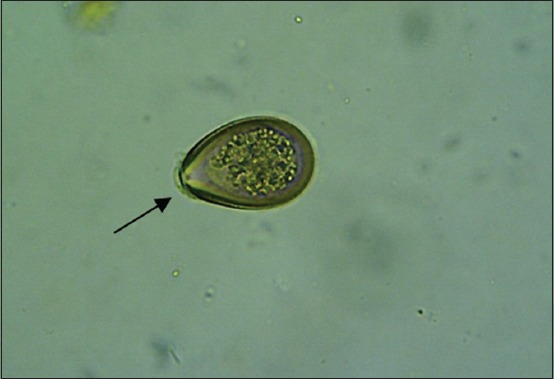
*Eimeria* (400×).

**Figure-5:**
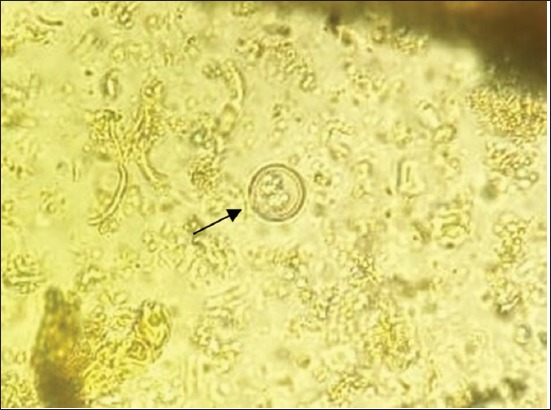
*Isospora* (400×).

**Figure-6:**
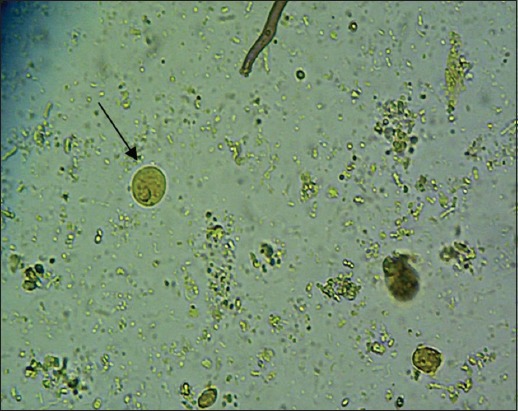
*Entamoeba* spp. (400×)

The results showed that there were six species of protozoa infecting the gastrointestinal tract of Madura cattle, i.e., *Eimeria* spp., *Balantidium* spp., *Isospora* spp., *Blastocystis* spp., *Entamoeba* spp., and *Cryptosporidium* spp. The result also indicated that one cattle could be infected by one, two, or even more three kinds of protozoa species at once (Table-2) (Figures-1-6).

*Eimeria* is the most common species of protozoan-infected Madura cattle in 10 districts, whether it was single or mixed infections. The second common species of protozoan-infected Madura cattle in Bangkalan district, both single and mixed infections, was *Blastocystis*. However, *Blastocystis* was not found infecting cattle in Arosbaya, Klampis, and Burneh districts.

Some types of protozoa found in this research showed to have zoonotic potentials, such as *Balantidium* spp., *Entamoeba* spp., *Blastocystis* spp., and *Cryptosporidium* spp. *Cryptosporidium parvum* oocyte is usually found in exoskeleton and gastrointestinal tract of *Musca domestica* on dairy farms. Flies as mechanical vectors have the ability to spread diarrheal diseases caused by protozoa as they can travel up to 20 miles [8]. *Cryptosporidium bovis* oocyte can infect six species of livestock and poultry in Tunisia [9]. The prevalence of *Cryptosporidium* infection in cattle in Northern Nigeria reported to be 22.3% [10,11], whereas the prevalence of *Blastocystis* spp., *Giardia* spp., and *Entamoeba* spp. was 14.6%, 12.45%, and 7.45%, respectively [12]. The high prevalence of *Blastocystis* spp. infection can lead to death in livestock [13,14]. The prevalence of protozoan infections in the gastrointestinal tract of dogs in France was 42.2%, and caused by *Blastocystis* spp. and *Cryptosporidium* spp. Both can potentially trigger infections to their hosts [15]. Meanwhile, the prevalence of *Entamoeba bovis* infection was 36% and mostly found in cattle suffering from diarrhea [16].

The number of samples with single infection caused by *Eimeria* spp. was 211, in which the highest was found in Socah district with 35 samples. Single infection caused by *Blastocystis* spp. was found in 57 samples and found at seven districts, in which the highest was found in Kamal and Labang districts, with 14 samples of each. Samples with single infection caused by *Balantidium* spp. was 26, in which the highest one was found in Burneh district with 18 samples. Single infection caused by *Entamoeba* spp. only found in one cattle in Labang district.

Samples infected by two species of protozoan, such as *Eimeria* and *Blastocystis*, were found in 34 at six districts. Sepuluh district with 11 samples was the highest. There were five samples infected with three species of protozoa (*Eimeria* spp., *Blastocystis* spp., and *Balantidium* spp.), which were found in Kamal district.

Based on sex, the prevalence of protozoan infections in bulls 65.54% (116/169) was lower than cows 72.97% (243/333). The comparison of the prevalence of protozoan infections based on sex in each district is shown in Table-3.

**Table-3:** The prevalence of protozoan infections in the gastrointestinal tract of Madura cattle in Bangkalan Regency based on sex.

Districts	Bulls		Cows		Total of samples	
		
	Number of samples	Positive samples (%)	Number of samples	Positive samples (%)	Number of samples	Positive samples (%)
Mangkon Arosbaya	13	6 (46.15)	38	32 (84.21)	51	38 (74.51)
Bator Ma’adan Klampis	10	8 (80)	30	24 (80)	40	32 (80)
Keleyan Socah	14	6 (42.86)	39	29 (74.44)	53	37 (67.28)
Gili Anyar Kamal	19	15 (78.95)	32	30 (93.75)	51	45 (88.23)
Maneron Sepuluh	23	19 (82.61)	30	27 (90)	53	46 (86.8)
Bumi Anyar Tanjung Bumi	22	17 (77.27)	26	19 (73.08)	48	36 (75)
Bringen Labang	4	3 (75)	48	32 (66.67)	52	35 (67.31)
Kemoneng Tragah	8	3 (37.5)	41	25 (60.98)	49	28 (57.14)
Ketengan Burneh	50	34 (68)	-	-	50	34 (68)
Kramin Bangkalan	4	3 (28)	49	25 (50.02)	53	28 (52.83)
Total	167	114 (68.26)	333	243 (72.97)	500	357 (71.4)

The prevalence of protozoan infections in cattle <6 months of age was 68.18% (45/66), with the highest case found in Klampis, Tanjung Bumi, and Burneh districts (100%), and the lowest was found in Sepuluh district (25%). Furthermore, the prevalence of protozoan infections in cattle aged from 6 months to 2 years was 73.39% (91/124). The highest prevalence was found in Arosbaya district (87.1%), and the lowest was found in Bangkalan district (23.08%). For the cattle >2 years, the prevalence of protozoan infections was 71.47% (223/312), the highest was found in Sepuluh district (94.12%), and the lowest was in Arosbaya district (52.63%) (Table-4).

**Table-4:** The prevalence of protozoan infections in the gastrointestinal tract of Madura cattle in Bangkalan Regency based on age.

Districts	Age of samples						Number of samples	Positive samples

	<6 months	Positive samples (%)	>6 months- 2 years	Positive samples (%)	>2 years	Positive samples (%)		
Mangkon Arosbaya	2	1 (50)	31	27 (87.1)	19	10 (52.63)	51	38
Bator Ma’adan Klampis	6	6 (100)	9	7 (77.78)	25	19 (76)	40	32
Keleyan Socah	17	12 (70.59)	10	8 (80)	26	15 (57.69)	55	37
Gili Anyar Kamal	2	1 (50)	18	15 (83.33)	31	29 (93.55)	51	45
Maneron Sepuluh	4	1 (25)	14	13 (86.67)	35	32 (94.12)	53	46
Bumi Anyar Tanjung Bumi	3	3 (100)	8	6 (75)	37	27 (72.97)	48	36
Bringen Labang	7	5 (71.43)	2	1 (50)	43	29 (67.44)	52	35
Kemoneng Tragah	10	8 (80)	2	-	37	20 (54.05)	49	28
Ketengan Burneh	1	1 (100)	16	11 (68.75)	33	22 (66.67)	50	34
Kramin Bangkalan	13	5 (38.46)	13	3 (23.08)	27	20 (74.07)	53	28
Total	65	43 (65.15)	122	91 (74.59)	313	223 (71.25)	500	357 (71.40)

Research conducted in Iran revealed that there were four types of protozoa species found in pigs with a prevalence of 64% [17], while the prevalence of protozoan infections in the gastrointestinal tract of carnivores in Iran was 80.4% [18]. Another research in India showed that 83.08% of cattle were infected by endoparasites, with the higher risk factor found in cows (85.97%) than bulls (69.23%) and in the adult ones that aged >6 months (85.97%) compared to those that aged <6 months (61.17%) [19]. Similarly, the prevalence of protozoan infections in cows in this research was also higher than in bulls due to predisposing genetic factor, hormonal factor, stress-reducing immune factor to infection, parturition factor, and lactation factor, resulting in weakness and malnutrition.

The prevalence of protozoan infections in Madura cattle in this research that aged from 6 months to 2 years was also higher than those aged <6 months due to their grazing habit in larger areas that were contaminated with protozoa. Hence, the prevalence of the infections in calf is always lower, since they were less exposed to larger areas contaminated with protozoa and it is also because the calf is still suckling.

Madura cattle are reared in cages or not, vulnerable to protozoan infection that contaminate the grass they eat. If one of the cattle was infected by protozoa, the area then would be considered as a good place for various types of protozoa to develop. Forages that were given to Madura cattle actually have been through the process of forging; however, the prevalence of protozoan infection in the gastrointestinal tract is still high because their feed and water have been contaminated by protozoa.

Rainy season that increased the humidity is also suspected to be the cause of the high protozoan infections in the gastrointestinal tract of Madura cattle. In other words, rainy season with high humidity and low temperatures is a condition favored by protozoa to thrive. Parasitic infections in livestock were also affected by various factors, including geographical location, environmental conditions, cage quality, sanitation and hygiene, cage density, temperature, humidity, as well as vegetation [6,20].

In this study, management of cage and sanitation is also known to be unfit since feces that were cleared out from the cages were dumped right around the cages, resulting in moisten up the cage which can elevate the risk of reinfection. The construction of the cages is also known to be very traditional and still not equipped with the feces and urine disposal line. The infections caused by one, two, and three types of protozoa species in this research may be caused by decreased immune system condition to resist against protozoan infection [3,20].

Poor ventilation system that can reduce sunlight to enter the cage will make the ground floor of the cage moist, so that it can be a good medium for parasitic growth. Pigs and cows in Korea are also known to be infected with *Balantidium* *coli* and *Entamoeba* spp., in which cysts and oocysts potentially pollute and contaminate their environment due to poor sanitation [21].

In addition, the data collected from the questionnaires indicated that most farmers were over 50 years old with low education (never attended school) or up to junior high school. Therefore, they had low understanding in receiving information as well as new ideas and technology to improve their cattle performance. High incidence of protozoan infection in this study indicates that gastrointestinal tract infections in Madura cattle were chronic and required treatment.

## Conclusion

The prevalence of protozoan infections on the gastrointestinal tract of Madura cattle was 71.51%. They were *Eimeria* spp., *Balantidium* spp., *Isospora* spp., *Blastocystis* spp., *Entamoeba* spp., and *Cryptosporidium* spp. About 58.76% samples were infected by one, 53.56% samples were infected by two types, and 2.19% were infected with three types of protozoan species. The prevalence of protozoan infections in cows and bulls was 72.97% and 65.54%, respectively. Based on age, the prevalence of protozoan infections in Madura cattle was 68.18%, 73.39%, and 71.47%, for 6 months, 6 month-2 years, and >2 years, respectively.

## Authors’ Contributions

PH, MD, and NDRL planned and designed the whole study. ES, LTS, and WMY collected sample, carried out the whole work, and wrote the manuscript. PH helped in identification of parasites and microscopic examination. MD, ES, and WMY helped during manuscript writing, cross-checking, and revision. All authors read and approved the final manuscript.
